# Modelling inflammation-induced peripheral sensitization in a dish—more complex than expected?

**DOI:** 10.1097/j.pain.0000000000003512

**Published:** 2025-02-25

**Authors:** Yuening Li, Amy Lock, Laura Fedele, Irene Zebochin, Alba Sabate, Matthew Siddle, Silvia Cainarca, Pascal Röderer, Katharina Montag, Paola Tarroni, Oliver Brüstle, Tanya Shaw, Leonie Taams, Franziska Denk

**Affiliations:** a Wolfson Sensory, Pain and Regeneration Centre (SPaRC), Guy's Campus, King's College London, London, United Kingdom; b Centre for Inflammation Biology & Cancer Immunology, Department of Inflammation Biology, School of Immunology & Microbial Sciences, King's College London, London, United Kingdom; c ULB Neuroscience Institute (UNI), Université Libre de Bruxelles (ULB), Gosselies, Belgium; d Institute of Liver Studies, School of Immunology & Microbial Sciences, King's College London, London, United Kingdom; e Axxam SpA, Openzone, Bresso, Milan, Italy; f Institute of Reconstructive Neurobiology, University of Bonn Medical Faculty & University Hospital Bonn, Bonn, Germany; g LIFE&BRAIN GmbH, Cellomics Unit, Bonn, Germany

**Keywords:** Pain, Inflammation, Stem cell–derived sensory neurons, IPSC, Calcium imaging, Gcamp, Patch clamping

## Abstract

Peripheral sensitization of nociceptors is believed to be a key driver of chronic pain states. Here, we sought to study the effects of a modified version of inflammatory soup on the excitability of human stem cell–derived sensory neurons. For this, we used a preexisting and a novel stem cell line, modified to stably express the calcium sensor GCamP6f. Upon treatment with inflammatory soup, we observed no changes in neuronal transcription or functional responses upon calcium imaging and only a very minor increase in resting membrane potential (RMP) via whole cell patch clamping: control RMP (−71.31 ± 1.1 mV) vs inflammatory soup RMP (−67.74 ± 1.29 mV), uncorrected 2-tailed independent samples *t* test, *P =* 0.0383. Similarly, small changes were observed when treating mouse primary sensory neurons with inflammatory soup. A semi-systematic reexamination of past literature further indicated that observed effects of inflammatory mediators on dissociated sensory neuron cultures are generally small. We conclude that modelling inflammation-induced peripheral sensitization in vitro is nontrivial and will require careful selection of mediators and/or more complex, longitudinal multicellular setups. Especially in the latter, our novel GCamP6f-induced pluripotent stem cell line may be of value.

## Abstract 2

Supplemental Digital Content is Available in the Text.

This manuscript assessed the effects of modified inflammatory soup on human stem cell–derived and primary mouse sensory neurons, using RNA-sequencing, patch clamping, and calcium imaging.

## 1. Introduction

Peripheral sensitization is the process by which sensory neurons alter their firing properties upon inflammation or injury, becoming more easily triggered in response to their usual ligands and activators.^14,39^ The latter include hot or cold temperatures, noxious mechanical activation, low pH, damaging chemicals like capsaicin, and endogenously produced proinflammatory molecules, like cytokines and growth factors. Given the importance of peripheral sensitization in a wide variety of painful conditions, including neuropathies,^26^ arthritis^50,52,64^ and immune-mediated inflammatory diseases,^24^ it is not surprising that many have tried to model it in an in vitro setting.

Early seminal studies by Handwerker, Reeh, and their teams used rodent skin-nerve explants to study the effects of what they called “inflammatory soup” (IS)—4 putative proalgesic molecules known to be present in an inflammatory environment: histamine, bradykinin, serotonin (5-HT), and prostaglandin-E2 (PGE2). They were able to demonstrate increased nociceptive neuron firing in the presence of IS, which was significantly potentiated by low pH.^58^ They also went on to show that IS causes pain when perfused through the skin of healthy volunteers.^57^

Since then, IS and its components have also been studied in dissociated dorsal root ganglion (DRG) and trigeminal ganglion (TG) cultures, where they are reported to alter neuronal hyperexcitability.^7,30,46,55,65^ Most of the work has been conducted in rodent cells with a few notable exceptions.^19,67^

Here, we set out to study the effects of IS in human-induced pluripotent stem cell (iPSC)-derived sensory neurons (iSNs). We used the Chambers protocol^10^ for differentiation with small molecules and a modified version of the original IS, adding tumor necrosis factor alpha (TNF) and nerve growth factor (NGF), in addition to 5-HT, bradykinin, histamine, and PGE2. This was to ensure that our inflammatory solution would contain no shortage of molecules, which can bind iSNs directly. For example, RNA sequencing (RNA-seq) suggests that bradykinin receptors are only expressed at very low levels in human postmortem DRG^27^ and absent from our iSNs (see Results). Similarly, PGE2 can bind 4 different receptors, 2 of which are clearly expressed in postmortem DRG (PTGER3 and prostaglandin E2 receptor 4 [PTGER4]). They are also the 2 that have been claimed to be most important for sensory neuron sensitization.^56^ In contrast, iSNs express only PTGER4 at rather low levels. Interestingly, the functional data on these 2 molecules are inverse to their neuronal receptor expression: bradykinin has consistently been shown to be painful when infused into skin,^47,57^ while PGE2 reportedly does not induce pain beyond that associated with fluid injection,^12^ unless potentiated by low pH.^49^

We assessed the consequences of modified-IS on our human sensory neurons using RNA-seq, calcium imaging, and patch clamping. We found its effects to be surprisingly modest and propose that this might be a general issue: a reexamination of prior literature, as well as our own experiments in mouse DRG neuron cultures indicate that studying peripheral sensitization in dissociated cells may not be as straightforward as conventionally assumed.

## 2. Methods

### 2.1. Generation of GCaMP6f-induced pluripotent stem cell line

The iPSC-line UKBi013-A (https://hpscreg.eu/cell-line/UKBi013-A) was generated via Sendai virus reprogramming of peripheral blood mononuclear cells, obtained from a healthy male donor. The use and generation of iPSC lines was approved by the Ethics Committee of the Medical Faculty of the University of Bonn (approval number 275/08), and informed consent was obtained from the donor. The human UKBi013-A-GCamP6f (UKB) iPSC reporter line for functional testing was generated by genome editing using CRISPR/Cas9 technology. The region to be modified, the AAVS1 safe-harbor locus, was PCR-amplified from a genomic DNA sample of UKBi013-A and sequenced. Variations from the NCBI reference genome sequence were recorded and incorporated into the targeting vectors. The following constructs were designed and generated: (1) a Cas9 expression construct driven by the elongation factor (EF) 1α promoter, (2) single guide RNA-expressing constructs specific for the targeted locus under control of a U6 RNA polymerase promoter, and (3) a homology-directed repair (HDR) donor plasmid, AAVS1-GCaMP6f, containing GCaMP6f under the cytomegalovirus immediate-early enhancer/ chicken beta-actin (CAG) promoter, as well as a puromycin selection cassette, flanked by locus-specific homology arms. In silico analysis of guide RNA off-target effects was performed to choose a sequence that does not have any predicted affinity for coding or exonic regions. Based on this, the following guide RNA was selected (GTCACCAATCCTGTCCCTAGTGG), with all of the top 20 predicted off-target sites additionally having at least 4 mismatches. All constructs were verified by sequencing and positively tested in proof-of-concept studies in HEK293 cells. To commence gene editing, the human iPSC line UKBi13A was then cultured and maintained in StemMACS iPS-Brew (Miltenyi Biotec, Germany, #130-104-368) and on Geltrex (Thermo Fisher Scientific, Europe, #A1413301) coated plates. Cells were passaged every 3 to 4 days using 0.5 mM EDTA. Single cell suspensions for transfection experiments were obtained after enzymatic treatment with Accutase (Thermo Fisher Scientific, #A1110501). Nucleofections were performed with a 4D-Nucleofector System (LONZA) using the Primary P3 kit according to the manufacturer's instructions (program used: CB-150). The following plasmids were cotransfected: the Cas9 expression construct, the AAVS1-targeting sgRNA expression construct, and the AAVS1-GCaMP6f HDR donor vector. After 48 hours of cell recovery, puromycin was applied to select for positive clones. After selection, individual clones were picked manually, expanded, and screened by PCR for positive modification of the AAVS1 locus. For positive clones, PCR products were sequenced and analysed to verify the correct editing of the targeted locus. Finally, the resulting edited line, UKBi013-A-GCamP6f, was subjected to high resolution SNP analysis to ensure that no chromosomal abnormalities could be detected.

### 2.2. Human-induced pluripotent stem cell–derived sensory neuron differentiation

Experiments were conducted with 2 iPSC lines derived from 1 healthy male and 1 healthy female human donor: HPSI0714i-kute_4 (Kute4), female origin, obtained via the Human Induced Pluripotent Stem Cell (HIPSCI) Initiative at King's College London and UKBi013-A-GCamP6f (UKB), male origin. Induced pluripotent stem cells were maintained on 6-well plates coated with vitronectin (StemCell Technologies, Europe, catalogue #100-0763) in Stemflex medium (Thermo Fisher, #A3349401) until 80% confluence. Cells were gently dissociated from wells using Versene (Thermo Fisher, #15040066) for 4 minutes at 37°C. Before differentiation, iPSCs were seeded on 6-well plates coated with Geltrex (Thermo Fisher, #A1413302) and allowed to expand in Stemflex medium to 60% confluence. At this point (day 1), the medium was changed to mouse embryonic fibroblast conditioned medium (MEF, Bio-Techne, United Kingdom, #AR005) supplemented with 10 ng/mL human recombinant FGF-2 (Miltenyi, #130-093-839). 24 hours later (day [D] 0), differentiation was initiated according to the Chambers protocol.^10^ Briefly, from D0 to D3, KSR medium (Knockout DMEM (ThermoFisher, #10829018), 15% knockout serum replacement (ThermoFisher, #10828010), 1% Glutamax (ThermoFisher, #35050038), 1% nonessential amino acids (ThermoFisher, #11140035), 100uM beta-mercaptoethanol (ThermoFisher, #31350010), 1% antibiotic/antimycotic (ThermoFisher, #15240096) was added and changed daily. From D0 to D1, SMAD inhibitors (10 µM SB431542, Bio-techne, #1614-10; 100 nM LDN 193189, Bio-techne, #6053-10) were added to medium. From D2 to D3, in addition to SMAD inhibitors, 3 µM CHIR99021 (Bio-techne, catalogue #4423-10), 10 µM SU5402 (Merck, #SML0443), and 10 µM DAPT (Bio-techne, #2634-10) were also added to medium. From D4 to D5, medium composition was changed to 75% KSR medium + 25% N2 medium (Neurobasal medium [ThermoFisher, #21103049]), 2% B27 supplement (ThermoFisher, #17504044), 1% N2 supplement (ThermoFisher, #17502048), 1% Glutamax, and 1% antibiotic/antimycotic) with all 5 small molecules included. From D6 to D7, medium composition was changed to 50% KSR + 50% N2, and only 3 µM CHIR99021, 10 µM SU5402, and 10 µM DAPT included. From D8 to D9, medium composition was changed to 25% KSR + 75% N2, and only 3 µM CHIR99021, 10 µM SU5402, and 10 µM DAPT included. On D10, medium composition was changed to 100% N2, and only 3 µM CHIR99021, 10 µM SU5402, and 10 µM DAPT included. On D11, immature neurons were dissociated from wells using TrypLE (ThermoFisher, #12604013) for 7 minutes at 37°C. Neurons were replated to Geltrex-coated 13-mm glass coverslips at a density of 75,000 cells/well. Neurons were matured and maintained in N2 medium containing 25 ng/mL human β-NGF (Peprotech, Europe, #450-01), 25 ng/mL human NT3 (Peprotech, #450-03), 25 ng/mL human GDNF (Peprotech, #450-10), and 25 ng/mL human BDNF (Peprotech, #450-02). From D11 to D14, the medium also contained 10 μM ROCKi (Enzo, United Kingdom, #ALX-270-333-M005) and 3 µM CHIR99021. The medium was changed twice a week. To remove proliferating, nonneuronal cells, 1-2 µM cytosine beta-D-arabinofuranoside (AraC, Scientific Laboratory Supplies, Europe #C1768-100 MG) was added, usually during the first (day 14) or second (day 17) medium change; depending on assessment of cell purity upon visual inspection, a second or third dose was added for certain differentiations. Each dose was followed by at least 1 medium change without AraC. AraC treatment was stopped 2 weeks before any functional studies and our days 50 to 70 RNA sequencing analyses. For the day 30 sequencing timepoint, cells were harvested 4 to 13 days after the last AraC dose. Neurons were matured for different lengths depending on each experimental question as described in Supplementary Table 1, http://links.lww.com/PAIN/C199 and the figure legends.

### 2.3. Mouse dorsal root ganglion cultures

All animal work was conducted in accordance with UK Home Office Legislation (Scientific Procedures Act 1986) and approved by the Home Office to be conducted at King's College London under a current project license. Cultures of dissociated DRG were prepared from male and female adult C56BL/6J mice purchased from Charles River. Mice were terminally anaesthetised with pentobarbital and transcardially perfused with 10 mL of ice-cold Dulbecco's phosphate-buffered saline (DPBS). Within 1 hour, as many DRG as possible were collected from cervical, thoracic, and lumbar levels. They were placed into dissociation buffer made up of F12 medium (Sigma-Aldrich, Europe, #N6658) containing 2.5 mg/mL collagenase (Sigma Aldrich, C9722) and 5 U/mL Dispase II (Sigma Aldrich, #D4693). After a 45-minute digestion at 37°C in a 5% CO_2_ incubator, DRG were triturated with a P1000 pipette and passed through a cell strainer. They were then resuspended in DRG medium (F12 with 1% antibiotic/antimycotic (Thermofisher, #15240096), 1% N2 supplement (ThermoFisher, #17502048), 10% FBS (Gibco, Europe, #10500-064) and plated at a density of 5000 cells per 24-well on poly-L-lysine (Sigma-Aldrich, #P8954) and Matrigel (Scientific laboratory supplies, #356230) coated coverslips. Patch clamp recordings were performed after 3 to 7 days in culture (see figure legends for more detail).

### 2.4. Immunostaining

Coverslips containing sensory neurons (days 46-81) were fixed in 2% paraformaldehyde (PFA) for 10 minutes at 37°C. They were rinsed twice with PBS and kept in PBS until staining. Coverslips were blocked for 1 hour in PBS-Triton (0.2% Triton X-100, Sigma-Aldrich, #T-9284) with 10% Normal Donkey Serum (NDS, Abcam, United Kingdom, #AB7475) at room temperature. They were then incubated at room temperature overnight with primary antibodies: 1:100 Brn3a (Sigma-Aldrich, #MAB1585) 1:500 PGP9.5 (Abcam, #ab108986), and 1:300 NeuN (Cell Signalling, United Kingdom, #12943S) in PBS-Triton + 10% NDS. On the second day, the coverslips were washed 3x with PBS before incubation with secondary antibodies (1:1000 donkey antimouse AF488 Invitrogen, 1:1000 donkey antirabbit AF568 Invitrogen, Europe) in PBS-Triton + 10% NDS for 2 hours at room temperature. They were then washed 3x in PBS and mounted on glass slides using DAPI-containing mounting medium (DAPI Fluoromount-G, #0100-20). The slides were left to dry overnight at room temperature before imaging.

### 2.5. Imaging and analysis

The slides were imaged using a Zeiss LSM 710 confocal microscope with a 20x objective. A minimum of 3 coverslips per trial were imaged. One representative picture per coverslip was analysed using FIJI,^53^ using a novel macro, which we have made freely available on Zenodo, alongside more detailed instructions (https://doi.org/10.5281/zenodo.12783430). Briefly, we first obtained a maximum intensity projection from the raw image. The channels were then split, and a binary mask of each channel was obtained using the “Threshold” function. The “Analyze Particle” function was used to calculate the total number of DAPI+ nuclei. All the channels were then analyzed in pairs using the “Colocalization Threshold” function followed by “Colour Threshold.” Because all the antibodies we used stained the nucleus (either exclusively or not), the resulting “regions of interest” (ROIs) represented the total number of colocalized nuclei detected. To obtain the final number of double positive nuclei, we excluded any ROI with area <30 µm to avoid any dead cells and background specks. Occasionally, 2 or more nuclei were counted by the programme as a single ROI; hence, we considered areas above 130 µm to be 2 nuclei and above 200 µm to be 3 joined nuclei.

### 2.6. Calcium imaging and analyses

Neurons derived from Kute4 or UKB lines over 50 days old were considered functionally mature and used in calcium imaging. The 50-day cutoff was based on our own sequencing results and on prior literature, which indicates that a minimum of 28 days in culture is needed to make mature cells,^28^ with papers generally indicating that longer maturation results in higher expression of sensory-neuron specific genes.^11,38^ Kute4 neurons were preincubated with 2.5 μg/μL Fura2-AM and 1uM probenecid (Sigma P8761) for 1 to 1.5 hours in an incubator (37°C, 5% CO_2_). For GCaMP6f+ UKB neurons, coverslips were habituated in extracellular solution (ECS) for about 1 hour before usage.

During the experiment, the coverslip was perfused with ECS containing: 140 mM NaCl (VWR, #7647-14-5), 5 mM KCl (Fisher Scientific, #7447-40-7), 10 mM glucose (Sigma, #G7021), 10 mM HEPES (Fisher Scientific, #BP310-500), 2 mM CaCl_2_ (Sigma, #C-7902), and 1 mM MgCl_2_ (BDH, #290964Y). The pH was adjusted to 7.4 with 1M NaOH. High KCl solution contained 95 mM NaCl, 50 mM KCl with the other ingredients identical, pH adjusted to 7.4. The perfusion speed was set to be around 4 mL/min. Pregnenolone sulphate (Sigma, #P162) and veratridine (ENZO, #BML-NA125-0010) were reconstituted according to the manufacturers' instruction and diluted in ECS to use at the final concentration indicated in the figures.

Calcium imaging was performed at room temperature on an inverted microscope (Nikon Eclipse TE200 microscope), equipped with a 10x air objective (NA 0.8) and a high-speed random-access monochromator (Photon Technology International) for the light source, and an ORCA-flash4.0 camera. To record Fura2 signal, measurements of 340 nm, 380 nm, and their ratio (340 nm/380 nm) were obtained by the Easyratio2 software. To record GCaMP6f signalling, measurements at 488 nm were obtained.

The time-lapse data were exported as videos in TIF files and then analysed in FIJI. To segment and select individual neurons, the StarDist plug-in Reference 54 was used to identify individual cells as ROIs followed by manual inspection. The measurement values at different frames were exported using the multimeasure function. Custom-made R code, made freely available on Zenodo (https://doi.org/10.5281/zenodo.13122856), was used to extract the mean value for each frame for each ROI. Specifically, ΔF/F was calculated by subtracting the reading for each time frame with the baseline average and divided by the baseline reading. A second baseline was calculated in the wash time just before 50 mM KCl. Live cells were defined from the ΔF/F value during the KCl period: at least 10 frames had to exceed the second baseline by at least 3 standard deviations (SD). Within the live-cell subset, PS and veratridine responders were defined as cells in which the ΔF/F values exceeded the baseline mean by at least 3 SD for a minimum of 80 data frames (∼2 minutes). The response also needed to occur within 5 minutes of the compound being added and be at least 10% above the baseline for a cell to be counted as a responder. The percentage of live cells and of drug responders was calculated. The max % response was obtained by averaging the 3 maximal values of drug responders over the response timeframe. Plots were created in R and Prism 9 GraphPad software.

Finally, unmodified UKBi013-A iSNs were differentiated until day 70, after which they were incubated for 30 to 45 minutes with a Fluo-4 NW Calcium Assay Kit (Invitrogen, #F36206) at 37°C. Cells were imaged using an InCell Analyzer 2200 plate microscope (GE Healthcare) at a frequency of 1 frame per second for 120 seconds. KCl was automatically added to the wells after a baseline of 20 seconds to reach a final concentration of 40 mM. The time-lapse images were analysed in FIJI and assigned a pseudo-color for better visualization.

### 2.7. Whole-cell patch clamp electrophysiology

Both iPSC-derived neurons and neurons dissociated from mouse DRG were continuously superfused with bicarbonate buffered solution containing in mM: 124 NaCl (Acros Organics, #207790250), 2.5 KCl (Thermoscientific, #196770010), 26 NaHCO_3_ (Sigma-Aldrich, #71631), 1 NaH_2_PO_4_ (Sigma-Aldrich, #71500), 2 CaCl_2_ (Honeywell Fluka, Europe, #21114), 1 MgCl_2_ (Honeywelll Fluka, #63020), 10 glucose (Sigma-Aldrich, #G8270), pH 7.4, bubbled with 95% O_2_ and 5% CO_2_. Patch pipettes solution consisted of (mM): 130 KGluconate (Acros Organics, Europe, #229322500), 2 NaCl (Acros Organics, #207790250), 0.01 CaCl_2_ (Honeywell Fluka, #21114), 2 MgCl_2_ (Honeywelll Fluka, #63020), 10 HEPES (Sigma-Aldrich, #H3375), 0.1 EGTA (Bio Basic, Canada, #ED0077), 2 ATP-Na (Sigma-Aldrich, #A7699), 0.5 GTP-Na (Sigma-Aldrich, #G8877), 10 Phospocreatine-Na (Sigma-Aldrich, #P7936), osmolarity 280 ± 10, adjusted to pH 7.2 with 1M KOH (BDH, #29627). The liquid junction potential was −14 mV and adjusted offline.

Dorsal root ganglion (3-7 days in culture) or iSNs (days 52-63 Kute4; days 45-55 UKB) were used for electrophysiological experiments. Cell culture medium for iSNs was left unchanged for 3 days before any experiments. This was to avoid any confounding effect of freshly added growth factors, eg, a medium change and thus acute application of a, albeit lower, dose of NGF already inducing neuronal sensitization^13^ even before modified-IS was added as part of an experiment. Coverslips were incubated for 18 to 26 hours with either control medium (Ctrl: DRG medium without FBS, N2 medium without growth factors) or modified inflammatory soup (modified-IS) medium (Ctrl medium with 100 ng/mL NGF [Peprotech, #450-01-100]), 100 ng/mL TNF (Peprotech, #300-01A-100), 1 μM histamine (Sigma-Aldrich, #H7250), 1 μM bradykinin (Sigma-Aldrich, #B3259), 1 μM serotonin (Sigma-Aldrich, #H9523), and 1 μM prostaglandin E2 (Bio-techne, #2296). All electrophysiological experiments were undertaken at room temperature. Patch pipettes were used at a resistance of 4.5 to 6.5 MΩ and were pulled using filamented borosilicate glass (Harvard Apparatus, North America, GC150F-10, #30-0057), with an outer and inner diameter of 1.5 mm and 0.86 mm, respectively. Whole-cell patch current clamp recordings were undertaken using a MultiClamp 700B amplifier (Molecular Devices), digitised using a Digidata 1440A (Molecular Devices), with an upright microscope Olympus BX51WI. Recordings were acquired and analysed with the pClamp software suite v.11.3. Five iSN differentiation batches were used with a total of 45 cells in each treatment group. Six mouse DRG cultures (3 males and 3 females) were used with a total of 26 to 29 cells in each treatment group.

Spontaneous action potentials were recorded in gap-free mode (2 minutes) at resting membrane potential. A cell was defined as firing spontaneous action potentials if it fired at least 1 action potential during this period. Evoked action potentials were recorded at a 0.5 Hz frequency using a 200 milliseconds current injection from −50 pA up to 850 pA with a stepwise 25 pA increase, both at resting membrane potential and after injecting currents to hold the cells −75 mV. Cells that did not evoke an action potential upon current injection (up to 850 pA) or with a resting membrane potential above −40 mV were excluded from our final analysis. Resting membrane potential was calculated as an average of the first 200 milliseconds before the stimulus protocol. Rheobase was measured at resting membrane potential and was defined as the minimum injected current that would elicit an action potential.

Action potential amplitude, threshold, half-width, and afterhyperpolarisation (fast and medium) were measured, holding cells at −75 mV. The peak amplitude was measured between the resting membrane potential (−75 mV) and the peak. Action potential threshold was defined using the first derivative method, when the membrane potential crossed dV/dt equal 10 mV/ms.^48^ Half-width is the duration of the action potential at half of its peak amplitude. Fast and medium afterhyperpolarization are, respectively, the minimum potential after the first action potential peak and the minimum potential after the stimulus protocol. Time to peak was measured as the time between the stimulus and the first action potential peak.

To assess the firing frequency (number of action potentials) upon current injection, an action potential was defined as such if dV/dt was equal or higher than 10 mV/ms.

### 2.8. RNA sequencing

Two batch-controlled sequencing experiments were conducted, comparing iSNs at 3 different timepoints (days 29-30, days 50-53, days 69-70) and comparing day 60 neurons treated with modified-IS vs control medium for 24 hours. For each group, 2 coverslips from a 24-well plate of neurons were combined into 350 µL RLT buffer from the RNeasy Micro Plus kit (Qiagen, #74134), supplemented with 1% beta-mercaptoethanol (PanReac AppliChem, Europe, #A1108.0100). The samples were stored at −80°C degrees, until batch-controlled RNA extraction, library preparation, and sequencing. RNA was purified using the RNeasy Micro Plus kit (Qiagen, #74134) following manufacturer's instructions. We quantified the RNA amount using a Qubit high sensitivity RNA assay (Invitrogen, #Q32851) and checked its quality using a Tapestation 4150 system (Agilent). All samples had a RIN value of more than 9.9. They were processed at once within the same library preparation and multiplexed into the same Illumina sequencing lane (150 bp, paired-end reads) by the service provider Novogene Sequencing.

Reads were pseudo-aligned with kallisto version 0.50.1 to the human genome: Homo Sapiens GCRh38, kallisto index version 13.^5^ An average of 32 M reads were sequenced per sample, of which an average of 28 M reads were successfully aligned. See Supplementary Table 2, http://links.lww.com/PAIN/C200 for alignment statistics for each sample. Genes were considered to be expressed in our dataset if their transcripts per million (TPM) value was equal to 1 or more in all samples of a given group. Differential expression was performed by running DESeq2^36^ in R. All fastq and processed files are available on the Gene Expression Omnibus (GEO) repository under accession number GSE268585.

Pseudobulk count data were obtained from previously published single-nucleus RNA-seq data^27,45^ using GEO accession numbers GSE168243 & GSE201586. The Jung et al.^27^ dataset was subset on their DRG neuron cluster, while the Nguyen et al.^45^ GEO deposition only contained the neuronal fraction of their sequencing data. In both cases, counts were normalised in Seurat v0.4^22^ using the “RC” method and summed across all nuclei within a sample. Here, we display data for all n = 6 separate samples in Nguyen et al. and n = 16 out of 18 samples in Jung et al., because 2 of the latter (original IDs: SAM24364375, SAM24364376) only contained sequencing data on 20 vs 6 nuclei each and thus were deemed too unreliable for pseudobulk generation. Please note that both datasets are somewhat contaminated with glial cell transcripts, like SOX10, COL15A1, and FABP7 detectable at varying levels. FABP7 ranked 6898th in Nguyen et al. & 233th in Jung et al. in terms of average expression across samples, while SOX10 and COL15A1 were closer to an average expression of 50 pseudobulk counts in both instances (range: 16-112). We estimate, based on prior knowledge about neuronal DRG expression, that pseudobulk counts of 50 or less are close to or within the noise range for these particular datasets.

### 2.9. Literature review with semi-systematic search

The following search string was used in Pubmed on 28 September 2023: ((inflammatory soup) OR (inflammation)) AND ((DRG) OR (nociceptors) OR (sensory neurons)) AND ((calcium imaging) OR (electrophys*)). It was designed to help retrieve as many past papers studying inflammation-induced peripheral sensitization in dissociated peripheral neuron cultures as possible. Abstract screening and data extraction was performed by our 3 first authors (A.L., Y.L., and L.F.). We included only studies that examined DRG or trigeminal ganglion neurons in response to an inflammatory agent or solution with either patch clamping or calcium imaging. Moreover, the work, including the neuronal sensitization step, had to have taken place in vitro, eg, we excluded experiments in which an inflammogen was injected into a mouse and differential excitability was subsequently investigated in dissociated cultures. The following information was extracted from papers which were included: which species was used and whether the cultures were dissociated DRG or TG; what proinflammatory stimulus was used; how long the treatment was applied; how many cells/coverslips were studied; what changes were reported in terms of patch clamp and calcium imaging parameters; and whether we could observe clear shortcomings in terms of design, reporting, and/or sample size. Raw results are provided in Supplementary Table 3, http://links.lww.com/PAIN/C201.

## 3. Results

### 3.1. Characterisation of induced pluripotent stem cell–derived sensory neurons

**Figure 1.**
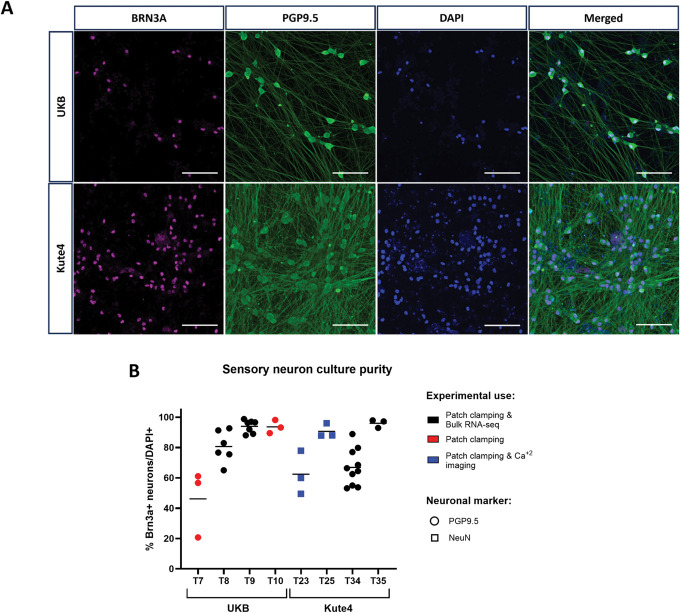
Immunostaining analysis demonstrates that pure sensory neurons could be differentiated from both our stem cell lines. (A) Representative images of UKB (day 54) and Kute4 (day 48) iPSC-derived sensory neurons. Scale bars: 100 µm. (B) Percentage of pure iSNs was determined for neurons from each independent differentiation used in subsequent experiments. Neurons aged days 46 to 81 were fixed and stained. Purity of iSNs was measured by taking the ratio of cells double positive for BRN3A and PGP9.5 or NeuN over DAPI+ nuclei. Each dot represents an individual coverslip. IDs T7-T35 refer to individual differentiations starting with UKB or Kute4 iPSC lines, respectively. iPSC, induced pluripotent stem cell; iSNs, induced pluripotent stem cell–derived sensory neurons.

We derived sensory neurons from 2 healthy human iPSC lines: Kute4 and UKB. UKB was modified from its originally founder line UKBi013-A to stably express the calcium sensor GCamP6f. After a well-established protocol,^10^ both lines were differentiated into sensory neuron cultures with high expression of sensory neuron marker BRNA3A and neuronal marker PGP9.5 (Fig. 1 and Supplementary Fig. 1, http://links.lww.com/PAIN/C199). All cultures were visually inspected via light microscopy to ensure viability, appropriate neuronal morphology and to exclude overt contamination by other cell types before subjecting them to further analyses. For most differentiations, we also set several wells aside for confirmatory immunofluorescent staining, which was quantified in Figure 1B. The results corroborated our “live” light microscopy assessments: there were both batch-to-batch and well-to-well differences, but the majority of differentiations yielded wells in which more than 90%+ of nuclei were BRNA3A positive, ie, the great majority of cells were sensory neurons.

**Figure 2.**
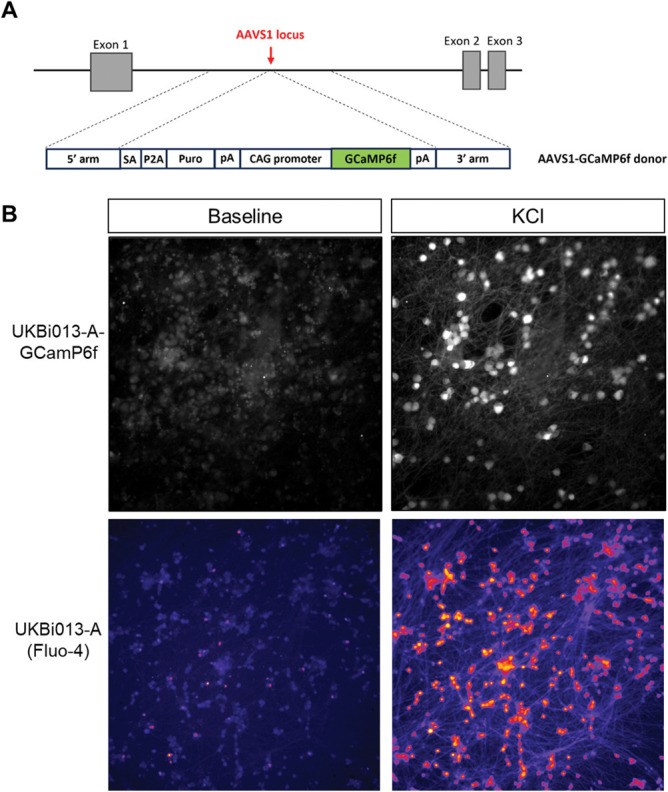
A novel iPSC line was generated, which constitutively expresses GCaMP6f. (A) Schematic overview of the genomic insertion of GCaMP6f in the AAVS1 safe harbour locus of the UKB line. The insert contained GCamP6f driven by the CAG promoter, as well as 3′ and 5′ homology arms, a 3′ splice acceptor site (SA), a P2A self-cleaving peptide sequence (P2A), and a puromycin resistance gene (puro) terminated by a poly-adenylation signal (pA). (B) Screenshots of UKB-derived iSNs during calcium imaging, illustrating GCaMP6f fluorescent signal before and after application of 50 mM KCl. For comparison, we also show screenshots of the UKB-line before it was modified, this time capturing calcium signal with the fluorescent dye Fluo-4 before and after application of 40 mM KCl. CAG, cytomegalovirus immediate-early enhancer/ chicken beta-actin; iPSC, induced pluripotent stem cell; iSNs, induced pluripotent stem cell–derived sensory neurons.

**Figure 3.**
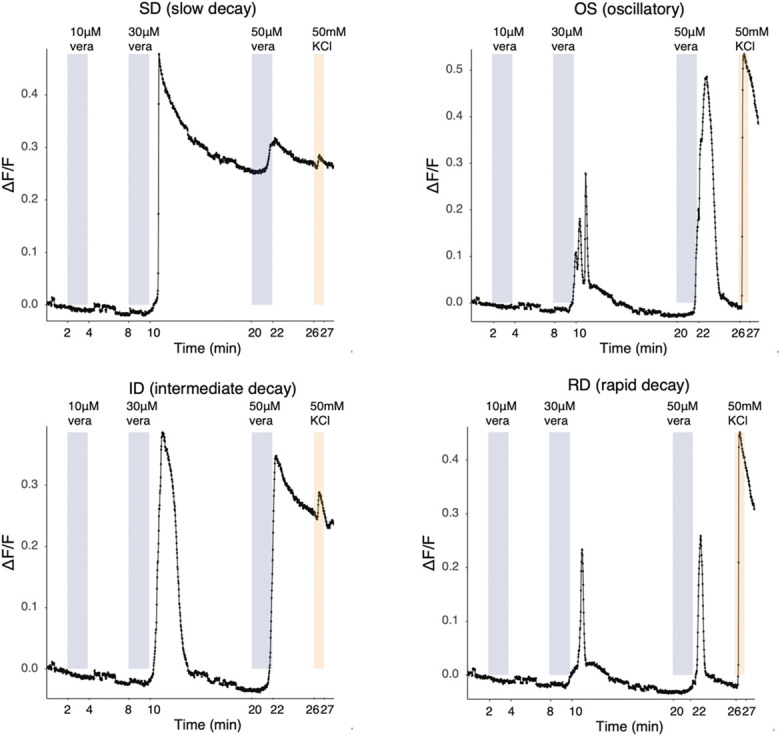
Representative traces of UKB-derived iSNs responding to veratridine demonstrating that the line's endogenous GCamP6f can be used to detect fast, dynamic responses. UKB iSNs respond to veratridine with distinct patterns, similar to mouse DRG neurons, as characterised by Reference 43. Neurons (82 days old) were subject to 3 different concentrations of veratridine. 50 mM KCl was used as a positive control. DRG, dorsal root ganglion; iSNs, induced pluripotent stem cell–derived sensory neurons.

As intended, calcium signals could be picked up directly in UKB-derived sensory neurons, with 50 mM KCl-induced depolarisation clearly visible when cells were excited at 488 nm (Fig. 2). Moreover, when neurons were stimulated with the sodium channel modulator veratridine, the fast dynamics of GCaMP6f enabled us to observe the expected 4 response patterns that have previously been described in mouse DRG neurons^43^ (Fig. 3 and Supplementary Video 1). Specifically, we recorded slow, intermediate, and rapidly decaying calcium transients, as well as ones with an oscillatory pattern; the latter 3 “transient shapes” are reported to be more common in small nociceptive neurons.^43^ We thus anticipate that the UKB iPSC line will be a useful tool for the functional study of human sensory neurons.

**Figure 4.**
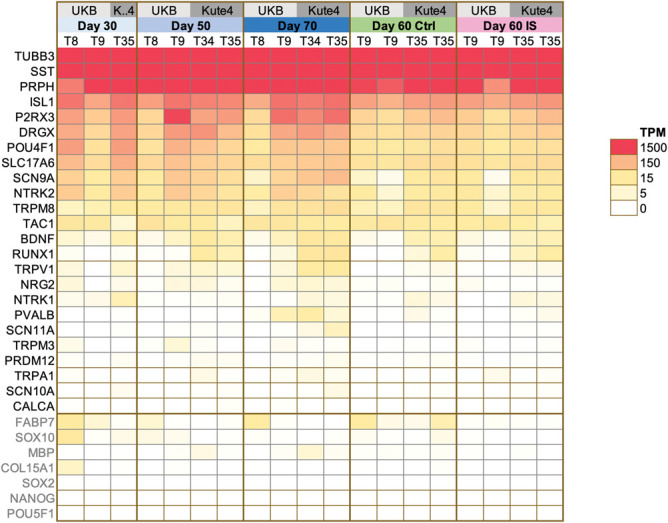
iSNs express common sensory neuronal markers, with low expression of nonneuronal markers. Heatmap showing TPM expression values for selected neuronal (in black) and nonneuronal markers (in grey). T8, T9, T34, and T35 are independent differentiations of 2 iPSC lines: UKB and Kute4, respectively. For the control and modified inflammatory soup conditions, day 60 neurons were treated for 24 hours before RNA was extracted. iSNs, induced pluripotent stem cell–derived sensory neurons; iPSC, induced pluripotent stem cell; TPM, transcripts per million.

To confirm the transcriptional identity of our iSNs and to better understand their maturation state, we bulk sequenced RNA from these neurons at days 29 to 30 (D30), days 50 to 53 (D50), and days 69 to 70 (D70) across 4 differentiations and 2 iPSC lines (Fig. 4 and Supplementary Tables 4 a–d, http://links.lww.com/PAIN/C219, http://links.lww.com/PAIN/C216, http://links.lww.com/PAIN/C217, http://links.lww.com/PAIN/C218). We found that iSNs across the 3 timepoints expressed common neuronal (TUBB3, PRPH), sensory neuron-specific (ISL1, POU4F1), peptidergic (TAC1) and nonpeptidergic (SST, P2RX3) markers. Pluripotency marker genes (NANOG, POU5F1) were fully downregulated. Finally, gene expression of markers for nonneuronal cell types (COL15A1 for fibroblasts, FABP7 for satellite glia, SOX10 & MBP for Schwann cells) was low or absent, confirming good purity of iSNs.

**Figure 5.**
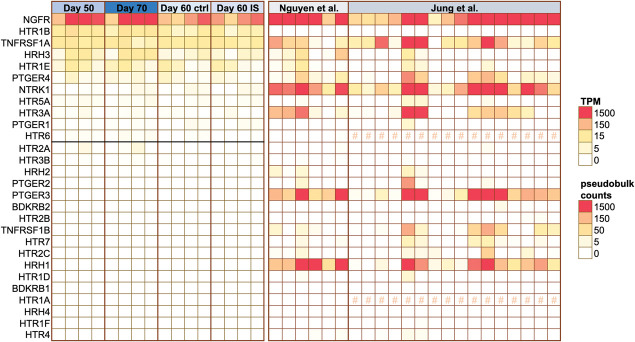
iSNs express receptors for several ligands within the modified-IS. TPM expression values for our iSN RNA-seq data are shown on the left. Each square is a sample, arranged in the same order as in Figure 4. All genes above the black horizontal line are expressed at TPM > 1 in at least 7 samples or more. Genes below the line fall within the noise range of our particular dataset. On the right, we plotted pseudobulk counts derived from human postmortem DRG, specifically single-nucleus RNA-seq data published by 2 independent groups (Nguyen et al.^45^ and Jung et al.^27^). Each square displays data on DRG neurons obtained from a different individual (n = 22 postmortem samples). We estimate that counts less than 50 are within the noise range. HTR1A and HTR6 were not annotated within Jung et al. and are, therefore, labelled with an #. DRG, dorsal root ganglion; IS, inflammatory soup; iSNs, induced pluripotent stem cell–derived sensory neurons; TPM, transcripts per million.

We were also able to confirm that our iSN model expresses receptors for several of the mediators present in our modified-IS (Fig. 5). NGFR (the low-affinity nerve growth factor receptor), HTR1B & HTR1E (2 of many serotonin receptors), TNFRSF1A (TNF receptor superfamily member 1A), and HRH3 (histamine receptor H3) were clearly expressed above threshold in all samples. The expression of the prostaglandin E2 receptor 4 (PTGER4) was at Transcripts per Million (TPM) > 1 in all but 1 sample, with an average of TPM of 3.9. NTRK1, the high affinity NGF receptor, was expressed at TPM > 1 in 8 out of 16 samples. When we compared these data to pseudobulk expression levels derived from 2 independent single-nucleus RNA-seq datasets of human postmortem DRG neurons,^27,45^ we identified important commonalities and differences. NGFR, PTGER4, TNFRSF1A, and HRH3 appeared comfortably expressed, similar to our iSN model system. However, in contrast to iSNs, HTR1E appeared more sporadically expressed and HTR1B seemed to be undetectable in all but 2 of 22 DRG neuron samples. Finally, NTRK1, PTGER3, HTR3A, and HRH1 were present in postmortem DRG samples, but less clearly expressed/absent in iSNs. Expression of these genes is highly likely to derive from neurons, although it is impossible to categorically rule out contamination in the case of PTGER3, HTR3A, and HRH1, because of the presence of glial and fibroblast-like transcripts in some of the postmortem DRG samples. Publicly available sequencing repositories suggest that none of these transcripts are likely to be found in Schwann cells or satellite glia^35^ (https://rna-seq-browser.herokuapp.com/), but PTGER3 and HRH1, for example, can be found in perineurial fibroblast-like cells, at least in mouse^20^ (https://snat.ethz.ch/search.html?q=trpa1).

**Figure 6.**
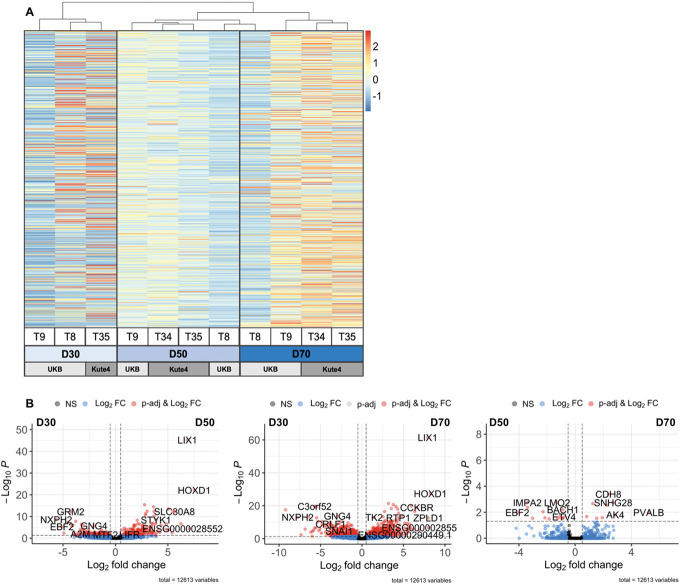
iSNs reach a consistent level of maturity beyond differentiation day 50. (A) Heatmap displaying differentially regulated genes across 3 iSN differentiation timepoints (D30, D50, and D70). Colours represent z-scored TPM values (by gene). Columns represent individual samples and were ordered using unsupervised clustering with the hclust function (method = ward.D2). Clustering was performed on all genes, which were expressed according to our cutoff (see Methods), differentially regulated at adj. *P* < 0.05 and regulated at log2FC > 1.5 or log2FC < −1.5. (B) Volcano plots displaying differentially expressed genes for each time point comparison. Adjusted *P* cutoff: <0.05, Log2FC cutoff: > 0.5 or < −0.5. Plots created using Enhanced Volcano R package.^4^ T = trial indicating independent differentiations of Kute4 and UKB lines. iSNs, induced pluripotent stem cell–derived sensory neurons; TPM, transcripts per million.

When comparing across timepoints, we found that D30 iSNs were the most distinct, with D50 and D70 samples clustering together (Fig. 6). Indeed, when comparing pairs of samples, D70 vs D30 had the highest number (949) of differentially expressed genes, followed by D50 vs D30 (312). Noticeably, only 23 genes were differentially expressed between D70 vs D50 (Supplementary Table 4c, http://links.lww.com/PAIN/C217). They include genes like PVALB and CDH8 (more abundant at D70) and BACH1 (more abundant at D50), suggesting some difference in sensory neuron phenotypes between the 2 groups. It is unclear whether this is a systematic, replicable difference denoting a further increase in maturity at D70, or whether it simply reflects the variability expected from small molecule iSN differentiations. Meanwhile, neurons at D30 are obviously different, with clear signs of immaturity, eg, lower expression of homeobox D1 (HOXD1), a known NGF-sensitive regulator of nociceptor circuitry in invertebrates,^21^ and limb-expressing 1 (LIX1), which has been linked to sensory neuron and nociceptor development. Specifically, Lix1 is reported to be significantly downregulated in Brna3-null^17^ and Islet-1 knock-out mice,^59^ both of which lack sensory neuron innervation.

### 3.2. Functional analysis of induced pluripotent stem cell–derived sensory neurons in response to modified inflammatory soup

**Figure 7.**
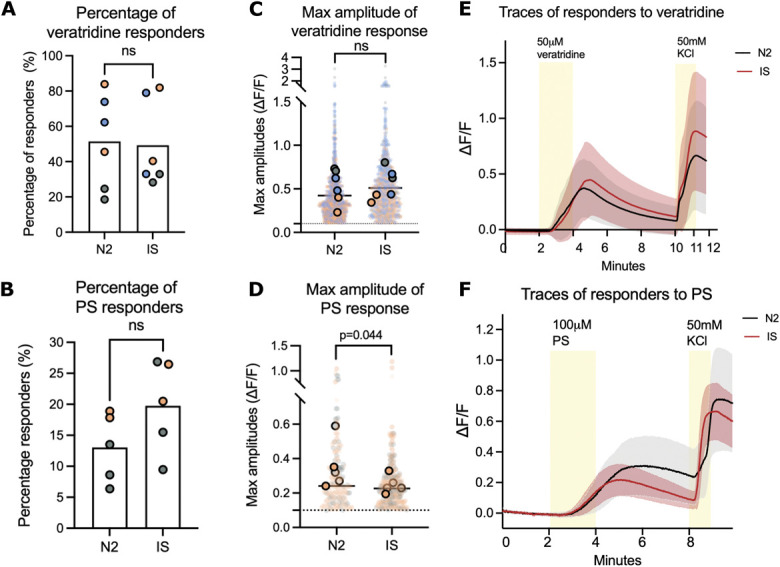
Modified-IS does not sensitise Kute4-derived iSNs in response to veratridine or pregnenolone sulphate (PS). Inflammatory soup does not change the response percentage (A and B) to 50 μM veratridine (n = 6 coverslips) or 100 μM pregnenolone sulphate (PS, n = 5 coverslips). Maximum response amplitudes were unchanged with modified-IS (C) or, if anything, reduced in response to PS (D). Each big dot in (A and B) represents a coverslip. In (C and D), the small, transparent dots are the maximal values of each cell in each coverslip. The superimposed large-sized dots are the mean values of each experiment. The dotted line is at 0.1, the cutoff for responders. Mann–Whitney nonparametric *t*-tests were used to compare between N2 and IS, with each coverslip taken as an independent unit (n = 6 for A and C, n = 5 for B and D). (E and F) Plot average traces in response to modified-IS and N2 control medium over the course of the experiment; the shades surrounding the average line are SD at each time point. For each condition, the trace is the average of 3 experiments. See Supplementary Figure 2, http://links.lww.com/PAIN/C199 for individual representative traces. For all experiments, neurons were incubated in N2 (medium control) or modified-IS for 24 hours before the experiment. Recordings took place at room temperature. Neurons were differentiated from Kute4 iPSC for at least 50 days. Different colours indicate independent differentiations (T23-29); for veratridine: grey = T28 72 days old; orange: T29 57 days old; blue: T29 62 days old; for PS experiments: grey = T25 63 days old; orange = T23 74 days old. iPSC, induced pluripotent stem cell; IS, inflammatory soup; iSNs, induced pluripotent stem cell–derived sensory neurons.

We next assessed the effects of modified-IS on sensitising iSNs via calcium imaging. After incubation in the soup for 24 hours, neurons (Kute4 iPSC, days 54-74) were subjected to 50 μM veratridine, which opens voltage-gated sodium channels or 100 μM pregnenolone sulphate, which activates transient receptor potential melastatin 3 (TRPM3) channels. TRPM3 has been implicated in peripheral sensitization^2^ and may convey information about noxious heat,^63^ although it has just been reported that blocking TRPM3 in humans does not affect heat pain ratings.^23^ Modified-IS did not appear to alter the percentage of cells that responded to either veratridine or pregnenolone sulphate (Figs. 7A and B). The magnitude of responses was also unchanged or, if anything, appeared somewhat reduced in the case of pregnenolone sulphate (Figs. 7C and D and Supplementary Fig. 2, http://links.lww.com/PAIN/C199). Notably, this result did not seem to be affected by the presence of nonneuronal cells: one of the trials (T23) was less pure than the other (T25) (Fig. 1B); however, both showed similar responses (grey vs orange dots in Figs. 7B and D). We were similarly unable to observe sensitization in response to veratridine when iSNs were treated with mod-IS vs vehicle acutely, for just 15 minutes (Supplementary Fig. 3, http://links.lww.com/PAIN/C199).

**Figure 8.**
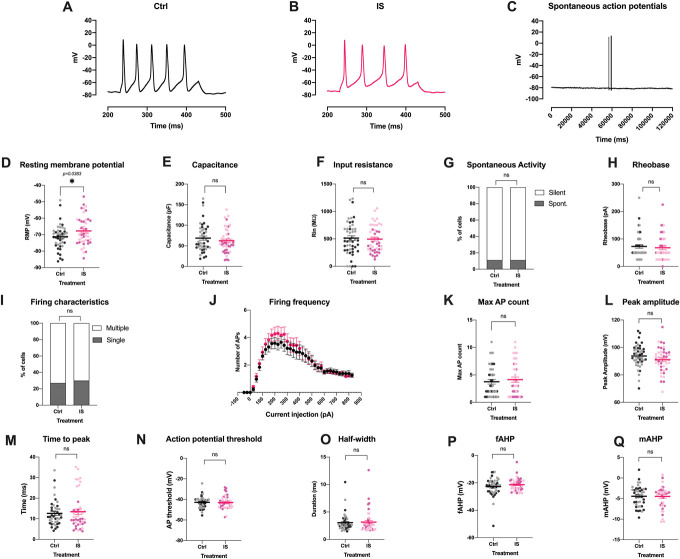
Modified-IS induces only a very minor change in resting membrane potential in iSNs, while all other electrophysiological properties of the neurons appear unaffected. Representative traces of evoked action potentials in control (A) and inflammatory (B) conditions and of spontaneous action potentials (C). (D) The resting membrane was more depolarised in modified-IS treated compared to control neurons (2 tailed unpaired *t* test, *P* = 0.0383). Modified-IS did not elicit any differences on capacitance (E), input resistance (F), spontaneous activity (G), rheobase (H), firing characteristics (I), firing frequency upon current injection (J), number of maximal action potentials (K), peak amplitude (L), time to peak (M), action potential threshold (N), half-width (O), fast afterhyperpolarisation (fAHP) (P), and medium afterhyperpolarisation (mAHP) (Q). Data were tested for normality and 2-tailed independent sample or Mann–Whitney *t*-tests were used accordingly for: (D, E, F, H, L, M, N, O). Fisher exact test was used to assess any potential changes in spontaneous activity and firing characteristics. Repeated measures mixed effects analyses were used to assess any changes in firing frequency between the 2 groups (J) with Bonferroni posthoc test: current injection resulted in significant changes in firing frequency (*P* < 0.0001); however, neither treatment (Ctrl vs IS) nor current injection × treatment was significant. All data represent mean ± SEM pooled from 5 different differentiations per line, n = 45 per treatment group. Darker colour represents Kute4 line, lighter colour UKB line. Ctrl: Kute4 n = 22, UKB n = 23; modified-IS: Kute4 n = 21, UKB n = 24. * *P* < 0.05. IS, inflammatory soup; iSNs, induced pluripotent stem cell–derived sensory neurons.

Traditionally, peripheral sensitization in a dish was most often assessed using direct measures of neuronal activity, rather than with indirect tools like calcium imaging. We, therefore, undertook whole-cell current-clamp recordings of iSNs in control conditions (Ctrl) or treated with modified-IS for 24 hours (Fig. 8). We had previously been involved in an effort where such recordings revealed increased excitability in mouse DRG nociceptors treated with conditioned medium from activated fibroblasts.^8^ This medium was shown to contain a cocktail of cytokines and proinflammatory molecules, although none that are within the modified-IS we used here. Nevertheless, we found that treating iSNs with modified-IS resulted in more depolarised resting membrane potential compared to the control group (2-tailed, independent samples *t* test, *P =* 0.0383, Ctrl: −71.31 ± 1.1 mV; IS: −67.74 ± 1.29 mV). In contrast, modified-IS did not have any effect on passive membrane properties of iSNs, with capacitance and input resistance very similar between the 2 groups. To study whether inflammatory soup changed any neuronal firing properties, we also assessed spontaneous action potential firing, rheobase, action potential firing characteristics, and frequency upon increasing current injection. The neurons from both groups behaved in a similar way regardless of the treatment. Finally, modified-IS did not result in any changes in evoked action potential properties, evident by the analyses of peak amplitude, time to peak, action potential threshold, half-width, fast afterhyperpolarisation (fAHP), and medium afterhyperpolarisation (mAHP).

**Figure 9.**
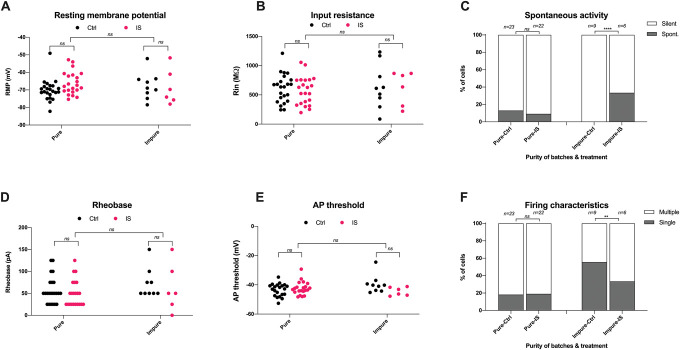
Differentiation batches that are less pure are not more likely to show signs of peripheral sensitization. Comparison of the effect of modified inflammatory soup on electrophysiological parameters reported from the literature between “pure” and “impure” iSNs. (A) Resting membrane potential, (B) input resistance, (C) spontaneous activity, (D) rheobase, (E) action potential threshold, (F) firing characteristics. Pure trials (T8, T9, T10, T25, and T35), Ctrl n = 23, mod. IS n = 22; impure trials (T7, T23 and T34), Ctrl n = 9, mod. IS n = 6. A 2-way ANOVA was used to compare groups displayed in (A, B, D and E), and a Fisher exact test was used for (C and F). IS, inflammatory soup; iSNs, induced pluripotent stem cell–derived sensory neurons.

These patch clamp data were generated using our 2 different iPSC lines over 5 independent differentiations. All neurons were at least 45 days old but varied in age range from 52 to 63 Kute4 and days 45 to 55 UKB. Yet, we could not detect any discernible patterns or differences between differentiations (Supplementary Figures 4 & 5, http://links.lww.com/PAIN/C199). We also examined whether the presence of nonneuronal cells might have impacted on neuronal excitability. For this, we grouped our iSN batches based on their purity as determined in Figure 1 and analysed our functional data accordingly. Differentiation batches were defined to be impure if the mean % of pure iSNs was below 70%; as such, T7, T23, and T34 were defined as “*impure”* and T8, T9, T10, T25, and T35 as “*pure*.” We specifically focused on electrophysiological parameters that have been identified by us or others in the literature to be modified when DRG neurons are treated with inflammatory stimuli (Fig. 9). Several differences could be observed at face value, specifically a more prominent increase in resting membrane potential in pure trials, a more obvious difference in spontaneous activity in impure trials, and a higher percentage of multiple action potentials in impure trials. The latter 2 observations were technically statistically significant. However, it remains unclear whether any of them would be replicable, given that our analyses were restricted to a low number of neurons in the impure group (n = 9 in ctrl and n = 6 in modified-IS).

### 3.3. Transcriptional changes in response to modified inflammatory soup

**Figure 10.**
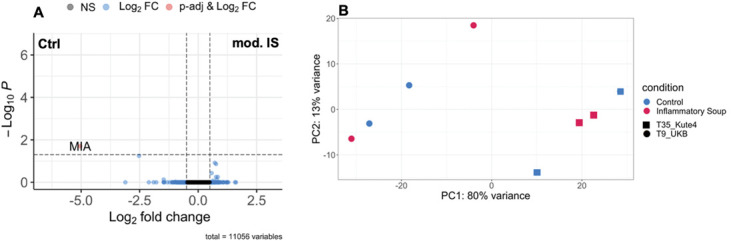
Incubation with modified-IS does not alter the transcriptome of iSNs. (A) Volcano plot displaying differentially expressed genes. Adjusted *P* cutoff: <0.05, Log2FC cutoff: > 0.5 or < −0.5. (B) PCA plots displaying points coloured by condition (control in blue and modified-IS in red) and shaped to indicate different iPSC lines (square for Kute4, circle for UKB). iPSC, induced pluripotent stem cell; IS, inflammatory soup; iSNs, induced pluripotent stem cell–derived sensory neurons; PCA, principal component analysis.

After our functional experiments using 24-hour incubation with modified-IS, we sought to investigate whether iSNs change transcriptionally under these conditions (Fig. 10). We used D60 iSNs from 2 differentiations, 1 from each iPSC line. Perhaps unsurprisingly, given the relative functional similarity between neurons with and without inflammatory mediators, a principal component analysis revealed samples to cluster by iPSC identity rather than treatment condition. Furthermore, differential expression analysis showed only 1 gene (MIA) significantly regulated at adj. *P* < 0.05, illustrating near zero transcriptional changes in response to modified-IS. No additional changes were revealed when each iPSC line was analysed individually (Supplementary Fig. 6, http://links.lww.com/PAIN/C199). MIA stands for melanoma-derived growth regulatory protein, which is expressed in satellite glia (https://rna-seq-browser.herokuapp.com/ and Ref. 25). It has higher TPM values in 2 control samples compared to the 2 other control and 4 mod-IS samples. The MIA-high control samples also have relatively higher expression of the canonical satellite glial cell marker FABP7 (Supplementary Table 4d, http://links.lww.com/PAIN/C218). The differential expression of MIA is, therefore, likely noise arising from a slightly higher nonneuronal cell content in these 2 wells.

### 3.4. Functional analysis of mouse dorsal root ganglion cultures in response to modified inflammatory soup

**Figure 11.**
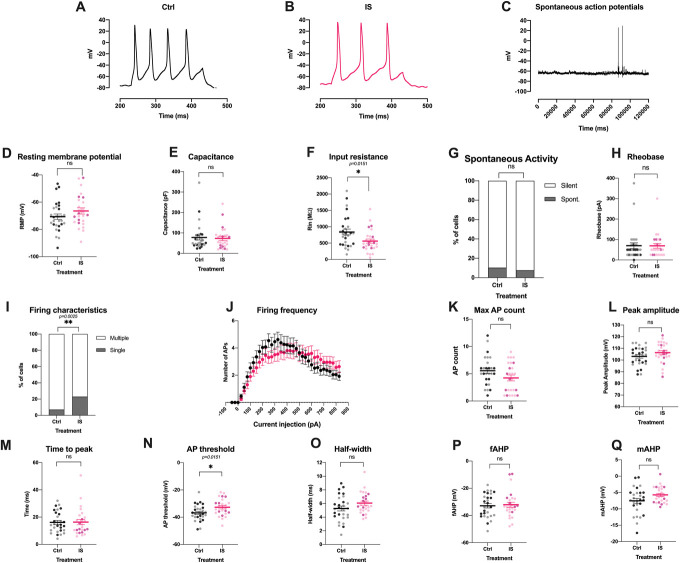
Modified-IS induces only very few, modest changes in the electrophysiological properties of mouse DRG neurons. Representative traces of evoked action potentials in control (A) and inflammatory (B) conditions and of spontaneous action potentials (C). mod-IS resulted in significant changes in input resistance (F) (Mann–Whitney test, *P*= 0.0153), firing characteristics (I) (Fisher exact test, *P* = 0.0025) and action potential threshold (N) (unpaired *t* test *P* = 0.0151). IS did not elicit any differences on resting membrane potential (D), capacitance (E), spontaneous activity (G), rheobase (H), firing frequency upon current injection (J), number of maximal action potentials (K), peak amplitude (L), time to peak (M), half-width (O), fast afterhyperpolarisation (fAHP) (P), and medium afterhyperpolarisation (mAHP) (Q). Data were tested for normality and two-tailed independent sample or Mann–Whitney *t*-tests were used accordingly (D, E, F, H, L, M, N, O). Fisher exact test was used to assess any potential changes in spontaneous activity and firing characteristics. Repeated measures mixed effects analyses were used to assess any changes in firing frequency between the 2 groups (J) with Bonferroni posthoc test: current injection resulted in significant changes in firing frequency (*P* < 0.0001) and current injection x Treatment (*P* = 0.0002); however, treatment alone (Ctrl vs IS) did not elicit any significant differences. All data represent mean ± SEM. DRG neurons were patched after 5 to 7 days in culture (Ctrl n = 26; IS n = 20) and after 3 days in culture (Ctrl: n = 4, IS n = 6), total Ctrl: n = 29, IS: n = 26. Darker colour represents cells isolated from male mice, lighter colour from female mice. *P < 0.05; **P < 0.01. DRG, dorsal root ganglion; IS, inflammatory soup.

Given that we observed only very modest effects in our iSN when treated with modified-IS, we wanted to confirm whether this could have been because of limitations of our stem cell–derived model system. In keeping with previously published sequencing data,^1,11,61^ our iSNs expressed only low levels of TRPV1 and the tetrodotoxin (TTX)-resistant sodium channel SCN10A (Fig. 4), 2 receptors known to be important for peripheral sensitization.^3,44^ Indeed, it has been reported that TTX-resistant currents in stem cell–derived sensory neurons more closely resemble those generated by Na_v_1.5, rather than Na_v_1.8 and Na_v_1.9.^16^ Moreover, we only very rarely found an iSN that was responsive to the TRPV1 agonist capsaicin (data not shown), which is in line with Chambers et al.'s originally reported response rate of 1% to 2%^10^ and the literature more generally.^31^ To test whether primary sensory neurons are more readily sensitized by modified-IS, we repeated our experiments in rodent cells. Mouse neurons treated with modified-IS for 24 hours displayed a small, but nonsignificant increase in resting membrane potential (Fig. 11). There was no effect on cell capacitance, but a reduction of input resistance (Ctrl: 839.35 ± 88.4 MW; IS: 561.967 ± 64.94 MW, 2-tailed Mann–Whitney test). One might speculate that reduction of input resistance without affecting cell capacitance denotes an increased number of open channels, possibly as a result of the action of inflammatory mediators on neurons. In terms of neuronal firing properties, modified-IS did not result in significant changes in the percentage of mouse neurons firing spontaneous action potentials nor did it change the rheobase or firing frequency upon current injection. However, we found that an increased number of cells in the modified-IS group fired single action potentials compared to control (2-sided, Fisher exact test *P =* 0.0025, *Ctrl* n *=* 29; IS, n *=* 26). We analysed the action potential shape and found that modified-IS resulted in a significantly more depolarised action potential threshold (Ctrl: −32.7 ± 2.58 mV; IS: −36.83 ± 1.11 mV, 2-tailed unpaired *t* test, *P =* 0.0151). However, none of the other parameters related to action potential shape were significantly different, including peak amplitude, time to peak, half-width, fast afterhyperpolarisation (fAHP), and medium afterhyperpolarisation (mAHP). Moreover, more single action potentials and an increased action potential threshold would indicate decreased excitability rather than the increased excitability one would predict with peripheral sensitization.

### 3.5. Review of the literature using a systematic search and qualitative analysis

**Figure 12.**
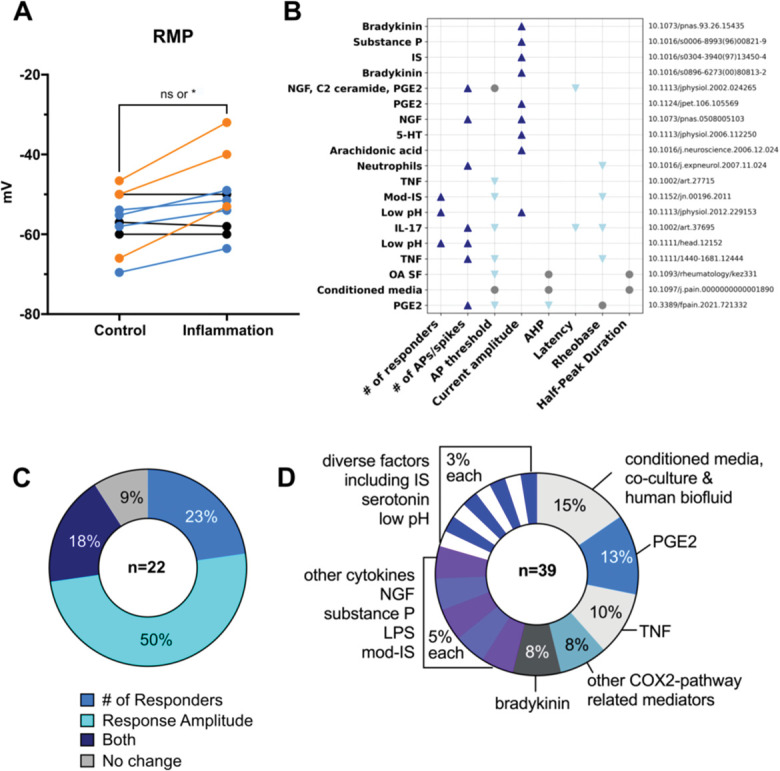
Review of previous literature indicates that mixed results are being reported in studies examining peripheral sensitization in primary dissociated cultures. Full data were extracted from a total of 39 studies; 10 measured the resting membrane potential (RMP) with the mean values reported in control wells and those treated with a substance designed to induce peripheral sensitization plotted in (A). Colours indicate effect sizes: 10 mV+ difference between group averages (orange), 2 to 6 mV difference between group averages (blue), 0 to 1 mV difference between group averages (black). When the 2 groups were analysed across all experiments with a nonparametric Mann–Whitney *t* test, no significant difference was detected (*P* = 0.27), when they were analysed with a paired parametric *t* test, the RMP was significantly increased in inflammatory conditions (*P = 0.0116). Nineteen studies conducted patch clamping, reporting changes in a variety of parameters plotted in (B). The blue up arrow indicates a study that reported an increase in a particular parameter, the light-blue down arrow indicates a decrease, and the grey circles indicate that no change was reported. Row labels represent the mediators used in each study, as well as their digital object identifiers. (C) Twenty-two studies conducted calcium imaging, reporting that peripheral sensitization induced changes in the number of responding neurons or in their response amplitude or both or neither. The substances used to induce peripheral sensitization in the 39 studies we extracted data from are summarised in (D). AP, action potential; AHP, afterhyperpolarization.

Finally, to aid interpretation of our data, we decided to compare our results to what has been reported previously in the literature. We undertook a semi-systematic search in Pubmed and screened 336 articles of which 59 were included in a data extraction step. Upon full-text screening, a further 8 were excluded because they did not use an inflammatory substance to treat the cells, while a further 12 were excluded because they only recorded specific channel currents rather than reporting on the more general action potential parameters we measured here. This left 39 studies from which we obtained quantitative data (Fig. 12). In terms of neuronal excitability, very few studies examined spontaneous activity (2 out of the 21 which conducted patch clamping). Instead, an increase in resting membrane potential was frequently observed (Fig. 12A), reported in 7 out of all 21 patch clamping studies (ie, 33%) and 70% of the 10, which specifically mentioned having recorded this particular neuron property. This is statistically significant at group level or not, depending on whether a more or less conservative *t* test is chosen for analysis (nonparametric Mann–Whitney vs parametric paired *t* test). Studies also reported many changes consistent with increased neuronal excitability, eg, decreased rheobase and action potential thresholds, increased numbers of action potentials or increases in current amplitude (Fig. 12B). However, it should be noted that most authors neglected to clarify how many parameters they examined in total; this makes it difficult to estimate the extent of contradictory results, eg, an increase in the number of action potentials in the absence of changes to the action potential threshold, as reported by Reference 68 and displayed in row 5 of Figure 12B. A total of 22 papers published calcium imaging data, 11 of which reported changes in amplitude, 5 of which observed changes in the number of responders, 4 of which changes in both, and 2 no changes at all (Fig. 12C). The most commonly used proinflammatory treatments were PGE2 (n = 5) or other COX2-pathway related substances (n = 3), TNF (n = 4), and proinflammatory supernatants or conditioned medium (n = 6), Figure 12D. Three studies examined inflammatory soup either in its original form (n = 2) or in a modification designed to mimic activated mast cells (n = 1). Inflammatory soup alone was reported to not be able to directly activate sensory neurons, unless applied under low pH conditions.^30^ To induce sensitization, most studies (n = 21) chose to apply inflammatory stimuli acutely (ie, within 10 minutes or less). However, there were n = 6 papers, which, like us, used 24 hours incubations (with inflammatory medium,^9,18,40^ bradykinin,^6^ a protein kinase C activator,^15^ or LPS^34^), and an additional n = 3 papers that used even longer incubation times of up to 84 hours (with inflammatory medium,^8^ PGE2,^55^ and NGF^37^). Finally, the great majority of studies had significant uncertainty associated with them, with only 10 papers deemed without clear limitations around experimental design, reporting, and/or sample sizes. For example, very low sample sizes were common, with only 4 to 9 neurons patched per condition. All relevant references and data associated with the search are provided in Supplementary Table 3, http://links.lww.com/PAIN/C201.

## 4. Discussion

This study set out to examine the response of human stem cell–derived sensory neurons (iSNs) to modified-IS. We observed no significant changes in calcium dynamics or transcriptional responses and only a very modest increase in resting membrane potential upon patch clamping.

There are several potential explanations for our results, which we will discuss in turn. First, in vivo, it is likely that nonneuronal cells play an important role in bringing about peripheral neuron sensitization, not just as sources of IS-components but also as intermediary cells, which may release additional proinflammatory mediators that bind nociceptors. For example, given known mRNA receptor expression patterns, it is likely that bradykinin exerts at least some, if not all, of its proalgesic effects via actions on, eg, keratinocytes.^42^ To test for the role of nonneuronal cells, we compared the response of our human iSNs between “purer” differentiations and those that were more contaminated with nonneuronal cells. We also performed patch clamping in conventional rodent DRG neuron cultures, dissociations of which contain a high proportion of nonneuronal cells,^60^ mostly made up of fibroblasts and a dedifferentiated mix of Schwann and satellite glial cells.^25^ We did not find any evidence that the presence of nonneuronal cells dramatically increases the extent to which sensory neurons respond to modified-IS in our dissociated in vitro settings.

As a second possible cause for the lack of sensitization, we focused on the inherent limitations of our iSN model system. Stem cell–derived sensory neurons are a great tool for the study of human peripheral nervous system function, but current differentiation protocols (the most commonly used of which is Chambers et al.^10^) fail to generate the full range of neuronal subclasses known to exist in vivo*.*^41,61^ In fact, rather than splitting into clear populations of A-fibres and peptidergic and nonpeptidergic C-fibres, iSNs tend to form 1 single homogeneous group of neurons that express some markers that usually do not co-occur (eg, SST and NF200^27^), while frequently expressing others, like TRPV1 and Na_v_1.8, at lower than expected and mostly nonfunctional levels.^16,31,61^ The latter 2 are particularly important in the context of peripheral sensitization, because phosphorylation of TrpV1,^44^ followed by regulation of TTX-resistant sodium channels like Na_v_1.8,^3^ is generally described as one of the key biological underpinnings of peripheral sensitization. Differentiations are also significantly affected by genotype,^33^ and our conclusions are limited by the fact that we only used 2 different iPSC lines. Yet, if our failure to detect abnormal excitability in response to modified-IS was purely because of a quirk within our human model system, we would have expected to detect clear signs of peripheral sensitization when patching mouse sensory neurons. After all, the concept of peripheral sensitization is best studied in rodent models, with many of the original publications on inflammatory soup and its constituents using dissociated cultures^7,30^ or skin-nerve preparations for electrophysiological studies.^29,58^

Overall, in our electrophysiological experiments, we examined 14 different parameters, which is not at all unusual for patch clamp experiments of this kind. This is noteworthy: historically, multiple comparison corrections would not have been routinely applied to such analyses, although, arguably, they are very necessary. Stringent correction methods, eg, Bonferroni, would indicate that we should apply a *P*-value threshold of 0.003 to reduce the chances of false positive results. We observed only a single *P*-value that was below this threshold, with modified-IS inducing more single than multiple action potential spikes in mouse DRG neurons. Yet, this result is the opposite of what one would expect from neurons that are more excitable because of peripheral sensitization. All other “significant” findings (increased resting membrane potential, reduced input resistance, and increased action potential threshold) were sporadic and did not replicate across species. This is also a pattern that is repeated across the literature: our semi-systematic review indicates that past papers frequently reported only 1 or 2 inflammation-induced alterations across a wide range of possible patch clamp parameters; crucially, it was usually unclear whether other parameters were not recorded or were unchanged and not written about. Additionally, single-cell electrophysiological techniques are inherently prone to unintentional p-hacking, because data are generated a few neurons at a time; with a series of experiments conducted over weeks or months, it is standard practice to plot some of the data “as you go along,” at which point one might decide to add more cells or to stop. This may seem intuitive, but statistically, this is a textbook example of sequential analysis, which requires controlling of type 1 error rates to avoid unintentional p-hacking^32^). All the above make it more likely that some of the previously reported effects are false positives or, at least, inflated in size.

This then, is a third possible explanation for the results we observed, and currently the one we deem most probable: modified-IS may simply not exert very large effects on the function of sensory neurons in primary monocultures—whether stem cell derived or dissociated from animals. Given what we know about the substances within modified-IS, this is probably less of a reflection of the proalgesic potential of individual components (shown to be high in in vivo settings) than a consequence of the highly artificial and reductionist environment of in vitro cell culture. The latter may lack key elements of an inflammatory environment, such as low pH or the presence of dysfunctional immune and/or stromal cells. Future studies may need to focus on increasing the effect size window in monocultures, eg, by using assays that allow for long-term recordings and subsequent within-cell comparisons, like multielectrode arrays. Further good options might be to use more complex, multicellular in vitro setups or other proinflammatory admixtures, eg, those modelling the function (and mediator release) of specific proalgesic cell subtypes, like activated fibroblast-like synoviocytes^8^ or Marco+ macrophages.^51^

In conclusion, here we have provided evidence to indicate that studying peripheral sensitization in dissociated cultures, whether human or murine in origin, is not straightforward. Even a mixture of well-known proalgesic mediators appears to induce only very modest functional effects in neurons, if any. In future, it will be important to optimise cellular model designs to more reliably demonstrate peripheral sensitization in vitro. We have provided a novel research tool that we hope will accelerate such endeavours, specifically an iPSC line, which constitutively expresses the calcium sensor GCamP6f. In parallel, more work is necessary to identify better-suited positive control substances; these should be uncovered by more systematic studies of the local inflammatory environment of chronic painful conditions, eg, to identify single mediators or, more likely, suitable combinations of mediators that nociceptors are exposed and can directly respond to. Human cohorts that examine peripheral tissues at cell type–specific level are currently being assembled at scale for many immune-mediated diseases, like rheumatoid arthritis^66^; yet, they frequently lack phenotyping data on pain perception.^50^ Meanwhile, efforts are being made to start collecting similar cohorts in the neuropathic pain field.^62^ Results from these various studies will help accelerate the search for a more reliable in vitro model of inflammation-induced sensory neuron abnormalities.

## Conflict of interest statement

O.B. is a cofounder, CEO, and shareholder of LIFE & BRAIN GmbH. None of the other authors have any conflicts of interest to declare in relation to this work.

## Supplemental digital content

Supplemental digital content associated with this article can be found online at http://links.lww.com/PAIN/C199, http://links.lww.com/PAIN/C200, http://links.lww.com/PAIN/C201, http://links.lww.com/PAIN/C219, http://links.lww.com/PAIN/C216, http://links.lww.com/PAIN/C217 and http://links.lww.com/PAIN/C218.

## Supplemental video content

A video associated with this article can be found on the PAIN Web site.

## Supplementary Material

SUPPLEMENTARY MATERIAL
